# Validity of new methods to evaluate renal function in cancer patients treated with cisplatin

**DOI:** 10.1007/s00280-016-2966-1

**Published:** 2016-01-20

**Authors:** Yohei Funakoshi, Yutaka Fujiwara, Naomi Kiyota, Toru Mukohara, Takanobu Shimada, Masanori Toyoda, Yoshinori Imamura, Naoko Chayahara, Hideo Tomioka, Michio Umezu, Naoki Otsuki, Ken-ichi Nibu, Hironobu Minami

**Affiliations:** Division of Medical Oncology/Hematology, Department of Medicine, Kobe University Hospital and Graduate School of Medicine, 7-5-2 Kusunoki-cho, Chuo-ku, Kobe, 650-0017 Japan; Division of Nephrology, Department of Medicine, Kobe University Hospital and Graduate School of Medicine, 7-5-2 Kusunoki-cho, Chuo-ku, Kobe, 650-0017 Japan; Department of Otorhinolaryngology - Head and Neck Surgery, Kobe University Hospital and Graduate School of Medicine, 7-5-2 Kusunoki-cho, Chuo-ku, Kobe, 650-0017 Japan; Cancer Center, Kobe University Hospital and Graduate School of Medicine, 7-5-2 Kusunoki-cho, Chuo-ku, Kobe, 650-0017 Japan; Division of Internal Medicine and Thoracic Oncology, National Cancer Center Hospital, 5-1-1 Tsukiji, Chuo-ku, Tokyo, 104-0045 Japan

**Keywords:** Cisplatin, Estimated glomerular filtration rate, Inulin clearance, Creatinine clearance, Cancer patients

## Abstract

**Purpose:**

Creatinine clearance (Ccr) is used as a marker of renal function in cancer chemotherapy, but it is not correlated with glomerular filtration rate (GFR) after high-dose cisplatin treatment. In addition to Ccr, measured using 24-h urine collection (24-h Ccr) or Cockcroft–Gault formula (CGF), the Chronic Kidney Disease Epidemiology Collaboration (CKD–EPI) equation and the Japanese GFR estimation equation (the Japanese equation) have been recently developed to estimate GFR for predicting renal function. However, these equations remain to be evaluated, particularly in cancer patients treated with cisplatin. Therefore, we investigated the validity of these equations for predicting the GFR in cancer patients treated with cisplatin.

**Methods:**

GFR was measured by inulin clearance (Cin) in 50 cancer patients and compared with GFR estimated by the CKD–EPI equation, the Japanese equation, and Ccr estimated by CGF or measured by 24-h Ccr before the first and third cisplatin-containing chemotherapy cycles (considered pretreatment and posttreatment, respectively).

**Results:**

Before treatment, the CKD–EPI and the Japanese equations estimated GFR with higher accuracy than Ccr. Posttreatment bias values for GFR estimation using the CKD–EPI and the Japanese equations were lower than those for Ccr. The CKD–EPI and the Japanese equations were also more precise than Ccr. However, for patients with low renal function, these equations still overestimated Cin.

**Conclusion:**

The CKD–EPI and the Japanese equations estimated GFR with lower bias and higher precision than Ccr pre- and postcisplatin treatment. This study is registered at UMIN: 000002167.

## Introduction

Cis-diamminedichloroplatinum(II) (cisplatin) is one of the most potent and valuable chemotherapy agents. Since its discovery over four decades ago, cisplatin has been widely used and has significantly improved the treatment of many malignancies, including head and neck, non-small cell lung, small cell lung, germ cell, and many other types of cancer [1–3]. However, cisplatin also causes adverse events including nausea, vomiting, peripheral neuropathy, ototoxicity, and nephrotoxicity [4, 5]. Among these, nephrotoxicity is the chief dose-limiting side effect, now that emesis can be well controlled by recently developed antiemetic therapies [6]. The mechanism of cisplatin nephrotoxicity is thought to be renal tubular damage by uptake into the S3 segment of the proximal tubule through the organic cation transporter-2 and the copper transporter CTR1 [7–10]. Because nephrotoxicity depends on the cumulative dose of cisplatin as well as on the dose per infusion, renal function decreases during repeated courses of cisplatin treatment. As urinary excretion is the main pathway for cisplatin elimination, accurate assessment of renal function is important for a safe administration of cisplatin and of other chemotherapeutic agents that are excreted in the urine [11, 12].

Glomerular filtration rate (GFR) is accepted as the most reasonable overall measure of renal function [13] and can be precisely assessed using filtration markers as inulin, ^125^I-iothalamate, ^51^Cr-ethylenediaminetetraacetic acid (^51^Cr-EDTA), or iohexol [13]. However, these techniques for the direct measurement of renal function are complex, expensive, time- and effort-consuming, and difficult to be performed in routine clinical practice or clinical trials of cancer chemotherapy. Instead, creatinine clearance (Ccr), measured using 24-h urine collection (24-h Ccr), or the Cockcroft–Gault formula (CGF), developed to estimate 24-h Ccr, has been used in daily practice and clinical trials [14]. However, assessment of renal function by Ccr overestimates GFR because creatinine is partly excreted via tubular secretion in addition to glomerular filtration in the kidney [15–18]; especially, when we apply these estimates using creatinine to cancer patients, it is important to note that creatinine production is influenced by muscle mass, nutritional state, and inflammation, which are often altered in cancer patients [13, 19–21]. Furthermore, it was reported that nephrotoxic anticancer agents, such as cisplatin, may alter the correlation between GFR and Ccr [22].

Daugaard et al. [22] suggested that Ccr was not suitable to measure renal function during high-dose cisplatin treatment (40 mg/m^2^ daily for 5 days) for germ cell tumors. After cisplatin administration, there was no correlation between GFR measured by ^51^Cr-EDTA and 24-h Ccr up to 3 months after the last dose of cisplatin because of progressive muscle wasting and tubular disorders caused by the drug. Therefore, although clinical trial protocols and empirical guidelines often require a normal Ccr for the administration of the full dose of cisplatin, it is unclear whether Ccr is a surrogate of GFR after administration of this drug, especially at a regular dose (70–100 mg/m^2^), which is widely used in chemotherapy in many cancers.

Recently, for better estimation of renal function in daily medical practice, the Chronic Kidney Disease Epidemiology Collaboration (CKD–EPI) equation has been developed from GFR measured by iothalamate [23] and is commonly used in the USA and Europe. Similarly, the equation derived from GFR measured by inulin clearance (Cin), namely the Japanese equation for estimated GFR (the Japanese equation), has been developed in Japan [24]. These equations for GFR estimation are more accurate than Ccr [24], and they have been widely accepted in medical practice. It is important to recognize that these equations were mainly developed in patients with chronic kidney disease (CKD), and cancer patients were not included in these populations. Furthermore, these equations also utilize creatinine, whose production is altered in cancer patients. Therefore, it is unclear whether these equations do accurately predict GFR in cancer patients. It is necessary to evaluate their validity for the estimation of renal function in cancer patients before they are implemented in oncology practice or clinical trials of cancer chemotherapy. Most importantly, the validity of these equations for the estimation of GFR in cancer patients during and after cisplatin treatment needs to be evaluated.

In the present study, to determine the most precise method for the estimation of GFR in cancer patients treated with cisplatin, we have prospectively assessed GFR by measuring Cin in Japanese cancer patients before the first and third cycles of chemotherapy containing cisplatin and compared Cin with GFR estimated using the CKD–EPI equation, the Japanese equation, CGF, and 24-h Ccr.

## Patients and methods

### Patient population

We investigated Cin in cancer patients hospitalized at the Kobe University Hospital receiving cisplatin-containing chemotherapy. Eligible patients (1) had histologically or cytologically confirmed cancer, (2) were scheduled to receive 70–100 mg/m^2^ of cisplatin (single dose), (3) had an expected survival of more than 3 months, (4) had adequate bone marrow and liver functions, (5) had the Japanese equation ≥50 mL/min/1.73 m^2^, and (6) had an Eastern Cooperative Oncology Group (ECOG) performance status of 0–1. Exclusion criteria were (1) concomitant medications, such as vitamin E or probucol, whose antioxidant activity affects the measurement of inulin levels, (2) contraindications to inulin, and (3) history of cisplatin administration within the previous 6 months. This study was performed in compliance with the Helsinki Declaration. All patients gave written informed consent to participate in this study, which was approved by the Institutional Review Board of the Kobe University Hospital.

### Evaluation of renal function

All estimations of renal function were performed within 7 days before the first and third cycles of cisplatin-containing chemotherapy. We measured Cin in the morning after overnight fasting, and serum and 24-hour urine samples for 24-h Ccr were obtained on the same day. We excluded from the analysis patients who did not collect all urine. Serum creatinine and inulin were measured by autoanalyzer using enzymatic methods. Cin was calculated from three sets of serum and urine inulin concentrations as well as urine volumes. Inulin was dissolved in physiological saline at a concentration of 1 % and infused intravenously over 2 h. The rate of infusion was 300 mL/h for the first 30 min and then 100 mL/h for the following 90 min. For the measurement of inulin concentrations, serum samples were collected at 45, 75, and 105 min into the infusion of inulin. After patients completely emptied the bladder at 30 min, urine samples were collected between 30 and 60 min, between 60 and 90 min, and between 90 and 120 min. GFR was predicted in each patient using the following formulae:

$$ \begin{aligned} &{\text{CKD}} {-} {\text{EPI}}\,{\text{equation }}\left( {{\text{mL}}/{ \hbox{min} }} \right)\left[ {23} \right] \hfill \\ &\quad = 141 \times { \hbox{min} }\left( {\frac{\text{Scr}}{\kappa },1} \right)^{\alpha } \times { \hbox{max} }\left( {\frac{\text{Scr}}{\kappa },1} \right)^{ - 1.209} \\&\quad \quad \times 0.993^{\text{Age}} \times \frac{\text{BSA}}{1.73}\left( { \times 1.018\,{\text{if}}\,{\text{ female}}} \right) \, \times 0.813 \hfill \\ \end{aligned} $$

where Scr is serum creatinine level. *κ* = 0.7 (0.9 if male), *α* = −0.329 (−0.411 if male), min = the minimum of Scr/*κ* or 1, max = the maximum of Scr/*κ* or 1.

The CKD–EPI equation was adjusted for Japanese patients by multiplying by 0.813 [25].

$$ \begin{aligned} & {\text{Japanese}}\,{\text{estimation }}\left( {{\text{mL}}/{ \hbox{min} }} \right) \, \left[ {24} \right] \hfill \\ &\quad = 194 \times {\text{Scr}}^{ - 1.094} \times {\text{age}}^{ - 0.287} \times \frac{\text{BSA}}{1.73}\left( { \times 0.739\,{\text{if}}\,{\text{female}}} \right) \hfill \\ \end{aligned} $$

Because the CKD–EPI and the Japanese equations estimate GFR adjusted for BSA, they were used after back-calculation to absolute values when correlation to Cin was investigated.

$$ \begin{aligned} & {\text{CGF }}\left( {{\text{mL}}/{ \hbox{min} }} \right)\left[ {14} \right] \hfill \\ &\quad = \frac{{\left( {140 {-}   {\text{age}}} \right) \times {\text{weight}}}}{{{\text{Scr}} \times 72}}\left( { \times 0.85\,{\text{if}}\,{\text{female}}} \right) \hfill \\ \end{aligned} $$

$$ \begin{aligned} & 24 \, {\text{h-Ccr}} \, \left({\rm mL/min} \right) \hfill \\ & \quad = \frac{{{\text{Ucr }} \times {\text{volume of }} {\text{24-h urine collection}}}}{{{\text{Scr}} \times 1440}} \hfill \\ \end{aligned} $$

where Scr is serum creatinine, BSA is body surface area, and Ucr is *creatinine* concentration in 24-h urine.

### Statistical analysis

All data were expressed as mean ± standard deviation (SD). A paired t test was used to investigate the statistical significance of differences between Cin and the CKD–EPI equation, the Japanese equation, CGF, and 24-h Ccr. *p* values <0.05 were considered to be statistically significant. Performance of the prediction of Cin by each formula was evaluated for bias and precision. Bias, a measure of systematic error, was expressed as both mean prediction error (ME) and mean percentage error (MPE); precision was shown as root-mean square error (RMSE), which was calculated as the square root of the sum of the squared error/*n*, where *n* is the sample size. The accuracy of prediction of the GFR by each formula relative to the Cin was expressed as percentage of the samples within 30 % of observed Cin.

Data from 50 patients were analyzed in this study. Assuming an *α* error of 0.05, a power of 90, and 30 % coefficient of variation for Cin and estimations of GFR by each formula, a sample size of 45 would detect a difference of 15 % between Cin and each estimate, which was considered clinically meaningful. IBM^®^ SPSS Statistics, version 19, was used to calculate statistical significance.

## Results

To obtain two data sets (pretreatment and posttreatment data) from 50 patients, 75 cancer patients who were scheduled to receive cisplatin-containing chemotherapy were enrolled in this study from July 2009 to December 2012 at the Kobe University Hospital. The two data sets could not be obtained from 25 patients because of discontinuation of cisplatin administration in seven (severe renal damage in two, other side effects in three and progressive disease in two), withdrawing consent in seven, technical errors in four, and difficulty in urine excretion in seven. The majority of the tumors were head and neck cancer (58 %), followed by esophageal (26 %) and lung (8 %) cancers (Table 1). On average, the total dose of cisplatin until the second measurement of renal function was 154 ± 19.2 mg/m^2^ (Table 1). None of the patients received other nephrotoxic drugs. The mean estimated GFRs by each method are shown in Table 2.

**Table 1:** Patients’ characteristics (*n* = 50)

	Pretreatment	Posttreatment
Sex (female, male)	16, 34	
Age (years), median (range)	64 (31–87)	64 (31–87)
Weight (kg)	56.0 ± 10.3 (32.6–78.8)	54.1 ± 10.1 (30.3–81.0)
BSA (m^2^)	1.59 ± 0.17 (1.19–1.98)	1.57 ± 0.17 (1.15–1.99)
BMI (kg/m^2^)	21.0 ± 2.87 (14.7–27.5)	20.3 ± 2.84 (13.7–27.0)
PS (0, 1, 2)	16, 34, 0	11, 33, 6
*Cancer type*		
Head and neck cancer	29	
Esophageal cancer	13	
Lung cancer	4	
Others	4	
*Regimen*		
CDDP + RT	17	
CDDP + 5-FU + RT	7	
CDDP + DTX	7	
CDDP + DTX + 5-FU	6	
CDDP + 5-FU	5	
Others	8	
Total dose of cisplatin (mg/m^2^)	154 ± 19.2 (112–200)	
*Complication* ^*a*^		
Diabetes	6	
Hypertension	12	

Mean ± standard deviation (range)

*BSA* body surface area, *BMI* body mass index, *PS* performance status, *CDDP* cisplatin, *RT* radiation, *5-FU* fluorouracil, *DTX* docetaxel

^a^The number of patients requiring medication

**Table 2:** Renal function (*n* = 50)

	Pretreatment	Posttreatment
Cin (mL/min)	76.7 ± 19.7 (35.6–142.3)	59.5 ± 22.1 (17.2–108.0)
The CKD–EPI equation (mL/min)	72.7 ± 12.5 (44.4–110.7)	66.7* ± 14.4 (30.6–111.3)
The Japanese equation (mL/min)	74.3 ± 17.3 (39.1–118.1)	66.0* ± 19.4 (30.9–132.4)
CGF (mL/min)	82.0 ± 25.5 (39.5–161.0)	72.4* ± 27.8 (30.2–176.1)
24-h Ccr (mL/min)	103.1* ± 32.8 (49.3–240.7)	78.8* ± 28.3 (23.7–153.6)
Serum creatinine (mg/dL)	0.72 ± 0.14 (0.44–1.00)	0.81 ± 0.21 (0.39–1.63)

Mean ± standard deviation (range)

*Cin* inulin clearance, *CKD–EPI* Chronic Kidney Disease Epidemiology Collaboration, *CGF* Cockcroft–Gault formula, *24-h Ccr* creatinine clearance from 24-h urine collection

* *p* < 0.05 versus Cin

### Pretreatment

**Fig. 1:**
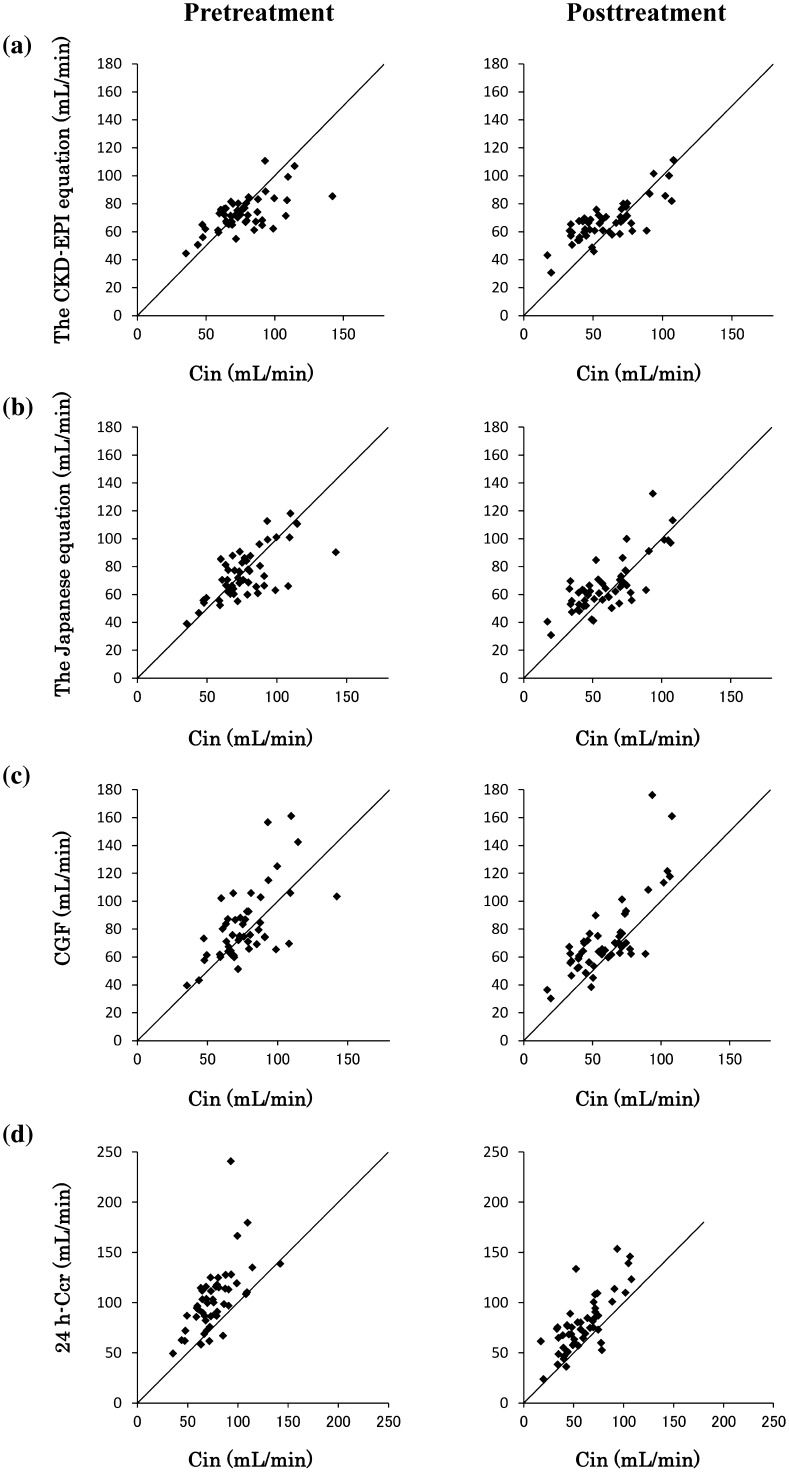
Relationship between inulin clearance (Cin) and **a** the Chronic Kidney Disease Epidemiology Collaboration (CKD–EPI) equation, **b** the Japanese glomerular filtration rate estimation equation (the Japanese equation), **c** the Cockcroft–Gault formula (CGF), and **d** creatinine clearance from 24-h urine collection (24-h Ccr). Black lines show the line of identity

Estimated values of GFR by the CKD–EPI and the Japanese equations were similar to Cin, but 24-h Ccr significantly overestimated renal function compared with Cin (Table 2). Bias (ME and MPE) of the CKD–EPI (−3.96 mL/min and −1.77 %) and the Japanese equations (−2.36 and −0.85 %) were approximately equal and smaller than CGF (5.38 mL/min and 8.61 %, *p* < 0.0001) and 24-h Ccr (26.4 mL/min and 36.1 %, *p* < 0.0001) (Table 3). When we compared precision (RSME) for the estimation of pretreatment Cin using the different approaches, the CKD–EPI and the Japanese equations were approximately equal (15.7 and 15.5 mL/min) and were more precise than CGF and 24-h Ccr (20.9 and 36.7 mL/min) (Table 3). Both the CKD–EPI and the Japanese equations were the most accurate for predicting GFR, with 92 % of the samples within 30 % of GFR (Table 3). When scatter plots of Cin against the other estimates were investigated before treatment, the CKD–EPI and the Japanese equations showed relatively good correlations even in cancer patients (Fig. 1a, b, left), whereas CGF and 24-h Ccr overestimated Cin in many patients (Fig. 1c, d, left).

**Table 3:** Bias and precision of prediction for Cin by each estimate

	Pretreatment				Posttreatment			
	ME (mL/min)	MPE (%)	RMSE (mL/min)	Accuracy within 30 % (%)	ME (mL/min)	MPE (%)	RMSE (mL/min)	Accuracy within 30 % (%)
The CKD–EPI equation	−3.96	−1.77	15.7	92	7.22	22.2	15.6	60
The Japanese equation	−2.36	−0.85	15.5	92	6.52	19.1	15.8	68
CGF	5.38	8.61	20.9	78	12.9	27.4	21.8	56
24-h Ccr	26.4	36.1	36.7	42	19.3	39.8	26.6	50

*ME* mean prediction error, *MPE* mean percentage error, *RMSE* root-mean square error, *CKD–EPI* Chronic Kidney Disease Epidemiology Collaboration, *CGF* Cockcroft–Gault formula, *24-h Ccr* creatinine clearance from 24-h urine collection

### Posttreatment

Weight, body mass index (BMI), and BSA in the two cycles of treatment were significantly lower than the respective pretreatment values (*p* < 0.05) (Table 1). Compared with pretreatment, posttreatment Cin significantly decreased from 76.7 ± 19.7 to 59.5 ± 22.1 mL/min (*p* < 0.05) (Table 2). Consistent with this decrease in Cin, serum creatinine was significantly increased after cisplatin treatment (0.72 ± 0.14 vs. 0.81 ± 0.21 mg/dL, *p* < 0.05) (Table 2).

After two cycles of chemotherapy with cisplatin, bias (ME and MPE) of the CKD–EPI (7.22 mL/min and 22.2 %) and of the Japanese equations (6.52 mL/min and 19.1 %) were approximately equal and smaller than CGF (12.9 mL/min and 27.4 %, *p* < 0.05) and 24-h Ccr (19.3 mL/min and 39.8 %, *p* < 0.0005) (Table 3). Similarly to the pretreatment, precision (RMSE) values of the posttreatment CKD–EPI (15.6 mL/min) and Japanese equations (15.8 mL/min) were also approximately equal and more precise than CGF (21.8 mL/min) and 24-h Ccr (26.6 mL/min) (Table 3). In the scatter diagram of the posttreatment values, CGF and 24-h Ccr overestimated Cin in most patients, as during pretreatment (Fig. 1c, d). In contrast, the CKD–EPI and the Japanese equations did not overestimate Cin, when Cin was normal. However, these equations also tended to overestimate Cin especially at decreased Cin levels (Fig. 1a, b, right). Accordingly, in patients with lower Cin (<50 mL/min), higher bias and lower precision values were obtained compared with patients with normal renal function (Cin ≥50 mL/min) (Table 4). Consequently, even if patients had a CKD–EPI equation value ≥60 mL/min, 26 % (9/35) of them had decreased renal function (Cin <50 mL/min). Similarly, 23 % (7/30) of the patients with a Japanese equation value ≥60 mL/min had a Cin <50 mL/min.

**Table 4:** Bias and precision of prediction for Cin using the CKD–EPI equation and the Japanese equation

	Posttreatment							
	<50 mL/min (Cin) (*n* = 20)				≥50 mL/min (Cin) (*n* = 30)			
	ME (mL/min)	MPE (%)	RMSE (mL/min)	Accuracy within 30 % (%)	ME (mL/min)	MPE (%)	RMSE (mL/min)	Accuracy within 30 % (%)
The CKD–EPI equation	19.0	53.8	20.4	15	−0.63	1.19	11.5	90
The Japanese equation	15.4	45.0	17.9	40	0.58	1.74	14.3	87

*ME* mean prediction error, *MPE* mean percentage error, *RMSE* root-mean square error, *Cin* inulin clearance, *CKD–EPI* Chronic Kidney Disease Epidemiology Collaboration, *CGF* Cockcroft–Gault formula, *24-h Ccr* creatinine clearance from 24-h urine collection

## Discussion

The evaluation of renal function in cancer patients is important for a safe chemotherapy. Renal function is always included in eligibility criteria for clinical trials, and it is repeatedly monitored during chemotherapy that includes nephrotoxic drugs such as cisplatin. However, it is unclear whether equations based on creatinine levels can be used as surrogates for GFR in cancer patients whose creatinine production has been altered by the disease. Furthermore, Ccr is not correlated with GFR after high-dose cisplatin administration [22]. Although the CKD–EPI and the Japanese equations are currently used as better estimates of GFR than Ccr, they were mostly developed in CKD patients without cancer. In the present study, we evaluated renal function in cancer patients before and after the administration of cisplatin. We found that 24-h Ccr and CGF overestimated GFR measured by Cin, regardless of cisplatin administration (Table 3; Fig. 1), probably because approximately 20 % of creatinine is cleared into the urine by proximal tubular secretion [15–17]. On the other hand, the CKD–EPI and the Japanese equations accurately estimated GFR even in cancer patients before cisplatin administration (Table 3; Fig. 1). These results are consistent with our previous report [26]. Furthermore, the new estimation methods, the CKD–EPI, and the Japanese equations predicted GFR with lower bias (ME and MEP) and greater precision (RMSE) than Ccr, not only before but also after cisplatin treatment (Table 3). Therefore, we recommend to replace Ccr with these new equations in cisplatin-containing chemotherapy. However, these equations tend to overestimate Cin in patients with low Cin levels after cisplatin administration (Table 4; Fig. 1). Even when patients appear to have good renal function (≥60 mL/min) according to the CKD–EPI or the Japanese equations, qualifying them for a repeated dose of cisplatin, approximately 25 % of them in fact have renal dysfunction (Cin <50 mL/min), thus requiring a reduction in the dose.

The best method for the estimation of renal function during treatment with nephrotoxic drugs such as cisplatin is controversial. While a significant decrease in measured GFR by ^51^Cr-EDTA has been observed after cisplatin treatment [22, 27, 28], serum creatinine and Ccr have not been reported to change in several studies [22, 29–31]. Among these reports, Daugaard et al. [22] noted that 24-h Ccr no longer correlated with ^51^Cr-EDTA clearance after high-dose cisplatin treatment. However, our study showed that Cin and 24-h Ccr correlated even after cisplatin treatment, although 24-h Ccr consistently overestimated Cin. First, the discrepancy between the two studies may be due to differences in the dose of cisplatin. In the previous report, a high dose of cisplatin (40 mg/m^2^ daily for 5 days) was used, whereas in our study we treated patients with moderate doses (70–100 mg/m^2^ as a single infusion), widely used in many cancers. Second, severe malnutrition leading to muscular atrophy might be responsible for the poor correlation in the previous study. Indeed, patients suffered from severe weight loss (an average of 10.35 ± 1.04 kg) during three cycles of chemotherapy [22]. In contrast, weight loss was small in our study, probably due not only to the lower doses of cisplatin but also to advances in antiemetic therapy. In our study, all patients received 5-hydroxytryptamine receptor antagonist or neurokinin 1 receptor antagonist [32], which were not available at the time of the previous study. Third, the number of patients in the previous study was small: renal function was evaluated after treatment in only 16 patients.

We revealed that the CKD–EPI equation and the Japanese equations estimate renal function more accurately than Ccr in cancer patients. However, chemotherapeutic regimens currently used in daily practice were established in clinical studies in the past when the new methods were not available, and we may not have to change daily practice for such regimens as long as toxicities of drugs that are excreted via the kidneys are at the expected levels. Therefore, further prospective studies are necessary to assess the efficacy and safety of cisplatin administration by using the new equations. Furthermore, we would like to propose to replace Ccr with the new equations to evaluate renal function in future clinical studies for developing new agents. Additionally, because other chemotherapeutic agents, including carboplatin and etoposide, are excreted in urine, dosing guidance should be evaluated by using the new equations.

In conclusion, lower bias and higher precision values were obtained using the CKD–EPI and the Japanese equations than using CGF and 24-h Ccr in cancer patients before and after chemotherapy with cisplatin. Therefore, it is recommended to use these new equations instead of Ccr for the evaluation of renal function when cisplatin-containing chemotherapy is used; however, it is important to note overestimate renal function in patients with low Cin.
